# Nanomaterials for Fighting Multidrug-Resistant Biofilm Infections

**DOI:** 10.34133/bmef.0017

**Published:** 2023-04-24

**Authors:** Vincent M. Rotello

**Affiliations:** Department of Chemistry, University of Massachusetts Amherst, 710 North Pleasant Street, Amherst, MA 01003, USA.

## Abstract

Multidrug-resistant bacterial infections represent a dire threat to global health. The development of antibiotic resistance in bacteria coupled with the lack of development of new antibiotics is creating infections requiring antibiotics of last resort, and even some infections for which we have no available treatment. Biofilm-based infections present some of the most challenging targets for treatment. The biofilm matrix provides a physical barrier that can impede access of antibiotics and antimicrobials to resident bacteria. The phenotypic diversity found in biofilms further exacerbates the difficulty of eliminating infections, with quiescent “persister” cells evading therapeutics and re-initiating infections after treatment. Nanomaterials provide a tool for combatting these refractory biofilm infections. The distinctive size regime and physical properties of nanomaterials provide them with the capability to penetrate and disrupt biofilms. Nanomaterials can also access antimicrobial pathways inaccessible to conventional antimicrobials, providing a synergistic strategy for treating biofilm infections. This review will summarize key challenges presented by antibiotic resistance and biofilms when treating infection and provide selected examples of how nanomaterials are being used to address these challenges.

## Introduction

Multidrug-resistant (MDR) bacterial infections present a global healthcare crisis [1]. The development of antibiotic resistance through antibiotic use and overuse has created infections that are difficult or impossible to treat using traditional small-molecule therapies [2,3]. The challenges presented by MDR bacteria are exacerbated when these bacteria form biofilms. The biofilm matrix provides physical protection of bacteria from the host immune system and many antimicrobials [4–6].The biofilm matrix also provides a heterogeneous environment that fosters phenotypic diversity in the resident microbes [7]. This diversity includes persister cells that have slow metabolic rates, with concomitant innate resistance to antibiotic targeting energy-dependent mechanisms [8].

Bacterial biofilms are present in many types of infections [9], including implants/joint replacements, circulatory infections (e.g., endocarditis), and bone infections [10,11]. Wound biofilm infections are a particularly acute therapeutic challenge [12,13]. The economic cost of these infections is high, costing $25B in the US alone [14]. Treatment of wound biofilm infections typically involves surgical removal of infected tissues (debridement) combined with an extended regimen of antibiotic therapy [15]. Surgery is both expensive and invasive, and the long-term administration of antibiotics can induce drug resistance [16].

Nanomaterials provide a unique toolkit for tailored interactions with biological systems [17,18]. Nanoparticles (NPs) can be fabricated to have unique physical properties, including optical [19,20] and magnetic behavior [21,22]. NPs can be fabricated to cover a size range commensurate with biosystems ranging from proteins to small bacteria. This size range, coupled with engineering of the NP surface, can be used to provide tailored and selective interactions with bacteria that can kill bacteria without harming mammalian cells [23,24]. Nanomaterials can also be engineered to penetrate and eradicate biofilms [25], providing the potential for a comprehensive strategy for combatting biofilm infections [26,27]. This review will discuss the challenges presented by biofilm infections, ground rules for NP-bacteria and biofilm interactions, and how nanomaterials can be used to address the challenge of MDR biofilm infections.

## A Brief Introduction to MDR Biofilm Infections

Microbial species have been in conflict for billions of years, competing for resources and preying on each other [28]. Over this time, microbial life has developed an array of offensive and defensive weapons and structures. One of the key weapons in their arsenal is the ability to biosynthesize small molecules capable of selectively killing other competing microorganisms [29]. This selectivity is useful to medicine, providing a subset of molecules that kill bacteria with reduced or no toxicity to mammalian cells. These biomolecules generally kill microbes through specific mechanisms targeting essential cellular processes. These small molecules are the basis for the antibiotics that are the current front line in fighting infections in the clinic.

**Fig. 1.**
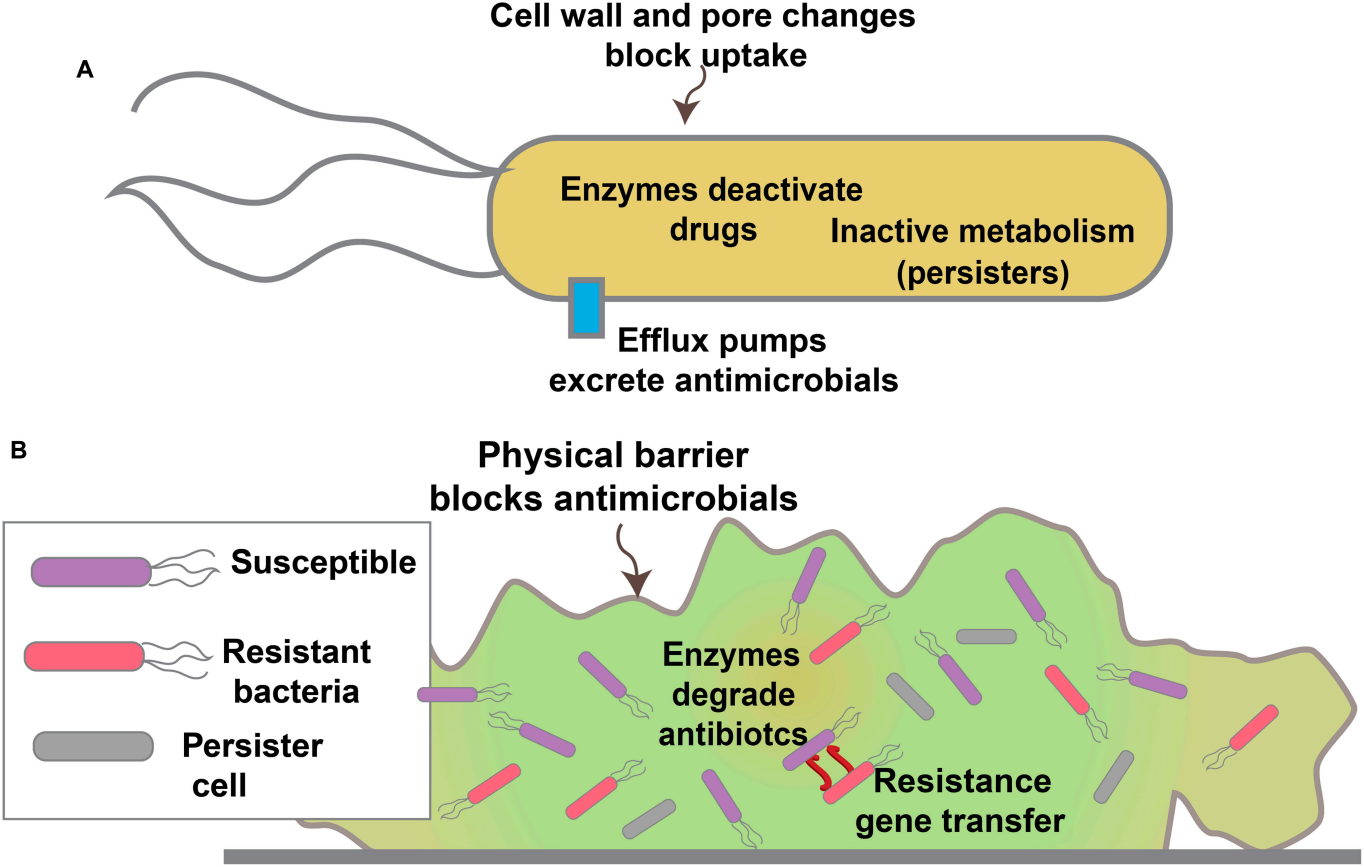
(A) Bacterial resistance strategies used by planktonic and biofilm bacteria. (B) Additional protection of bacteria afforded by biofilms, along with phenotypic variation (e.g., persister cells) to evade threats.

The fact that antibiotics are derived from microbial bioweapons means the microorganisms they target have likewise had billions of years to develop defenses against them [30]. There are 3 main defenses used by planktonic (dispersed) bacteria against antibiotics (Fig. 1A). First, bacteria have developed cell surface protection, for example, the bacterial outer membrane in the envelopes that protect Gram-negative bacteria from a number of antibiotics [31]. The second is enzymatic deactivation of the antibiotic, which can occur via a wide variety of enzymes including β-lactamases, esterases, and oxygenases [32]. The third strategy is the use of energy-dependent (ATP or sodium/proton gradient) efflux pumps that remove antibiotics from bacteria [33]. The rapid development of resistance in bacteria is facilitated by sharing of genetic information, spreading resistance genes across bacterial populations [30].

Building shelter can be an effective response to external threats. Biofilms present a defensive “fort” for protection of microbes against other microbes as well as environmental hazards (Fig. 1B) [34]. More than just protection, biofilms can harbor a complex and cooperative community. Bacterial biofilms are composed of bacteria embedded in a matrix of extracellular polymeric substance (EPS). There is an incredible array of biomolecular materials found in EPS, including proteins, polysaccharides, lipids, and DNA, with composition varying by species and strain [35]. This dense matrix provides a physical barrier that can protect the bacteria from external agents such as antibiotics through exclusion, diffusion, and dilution within the matrix [6]. Further, the proximity of bacteria in biofilms facilitates horizontal gene transfer that transmits resistance across the community.

Beyond protection, the biofilm provides a phenotypically diverse community that enhances microbial survival [36]. The heterogeneous structure of the biofilms creates regions where bacteria are isolated from nutrients and become quiescent. These metabolically slowed bacteria are called persister cells [37]. Most antibiotics target active processes in bacteria; hence, the lack of metabolic activity in persister cells provides a form of protection. These persister cells provide reservoirs of bacteria when biofilms are under attack, making their eradication difficult and promoting antimicrobial resistance [38].

## Engineering Nanomaterials to Kill Bacteria

The most important step in eradicating biofilm infections is killing of bacteria. Biofilm dispersion without killing would foster the spread of bacteria, potentially exacerbating the infection. Fortunately, nanomaterials provide an almost infinite array of platforms for the creation of antimicrobials [39]. The ability to control size, composition, and surface properties of NPs provides ample dimensions for engineering antibacterial activity through a wide range of mechanisms [40]. The most commonly used NP-based antimicrobial strategies include direct damage of cell walls and membranes, delivery of antimicrobials, generation of reactive oxygen species (ROS), and binding to intracellular machinery [23].

### Direct damage of cell walls and membranes

Integrity of the envelope (cell wall/membrane) surrounding bacteria is central to their survival. Disruption of this envelope provides an effective means of killing bacteria, and is the operative principle for a wide range of surfactant-based antimicrobials. The key challenge to applying this strategy therapeutically is obtaining sufficient selectivity for killing bacteria relative to the host (mammalian/human) cells. The difference in surface charge between bacteria and mammalian cells provides a route to selectivity. The surfaces of bacteria are overall more negatively charged than those of mammalian cells, making them more electrostatically attractive to cationic species [41]. Small-molecule cationic surfactants are widely used for cleaning surfaces. These surfactants, however, interact with membranes on a local level and are generally unable to discriminate between bacterial and mammalian cell surfaces based on surface charge. Natural products, including antimicrobial peptides, present multivalent surfaces that can provide bacterial/mammalian cell selectivity. Nanomaterials likewise present larger multivalent surfaces that can be engineered to provide selectivity against bacteria. In general, amphiphilic cationic NPs bind and disrupt bacterial membranes. The key feature in obtaining selectivity is control of charge and hydrophobicity: overly cationic or hydrophobic nanomaterials will have increased interactions with mammalian cells with concomitantly less selectivity against bacteria.

### Antimicrobial delivery

Nanomaterials provide versatile carriers for antimicrobials. NPs can protect cargo and improve solubility and stability of antimicrobials. Silver NPs are the most widely employed nanoformulation for antimicrobial use [42]. With these systems, the high surface area of the NP provides controlled dissolution to release antimicrobial silver ions. Small-molecule therapeutics can likewise be attached to or encapsulated inside NPs [43]. Responsive nanomaterials provide the capability of controlled release at infection sites, e.g., through antibiotic release in acidic infection sites [44].

### ROS generation

ROS can kill bacteria through a variety of mechanisms [45] with the most prominent pathway being through deactivation of membrane surface receptors via reaction of superoxide and hydroxyl radicals with thiols [46]. NPs can generate ROS through a variety of mechanisms. NPs can leach ions (e.g., Cu+) that generate ROS in bacteria [47]. NPs (in particular metallic) can generate ROS through excitation using light and other electromagnetic radiation, a photocatalytic process known as photodynamic therapy that can be particularly useful for wound treatment [48].

### Inactivation of cellular machinery

NPs can be engineered with sizes commensurate with proteins and nucleic acids, making them ideally sized for binding and disrupting intracellular processes. NPs have been used to interfere with gene expression and to bind intracellular proteins leading to killing of the bacteria [49].

## Penetration of Nanomaterials into Biofilms

The protected and diverse community presented by biofilms makes biofilm infections difficult to treat [15,16]. The ability of small molecules and nanomaterials to kill bacteria provides the first step in fighting biofilm infections. There remains, however, the key question of how to transport these antimicrobial agents into biofilms. Design strategy can be derived from the structure of biofilms. Bacteria are typically negatively charged, and the nucleic acids, polysaccharides, and proteins comprising the EPS are likewise rich in anionic and hydrophobic constituents. The biofilm matrix also features water-filled pores (≤350 nm in diameter) that allow nutrient transport into biofilms [7].

**Fig. 2.**
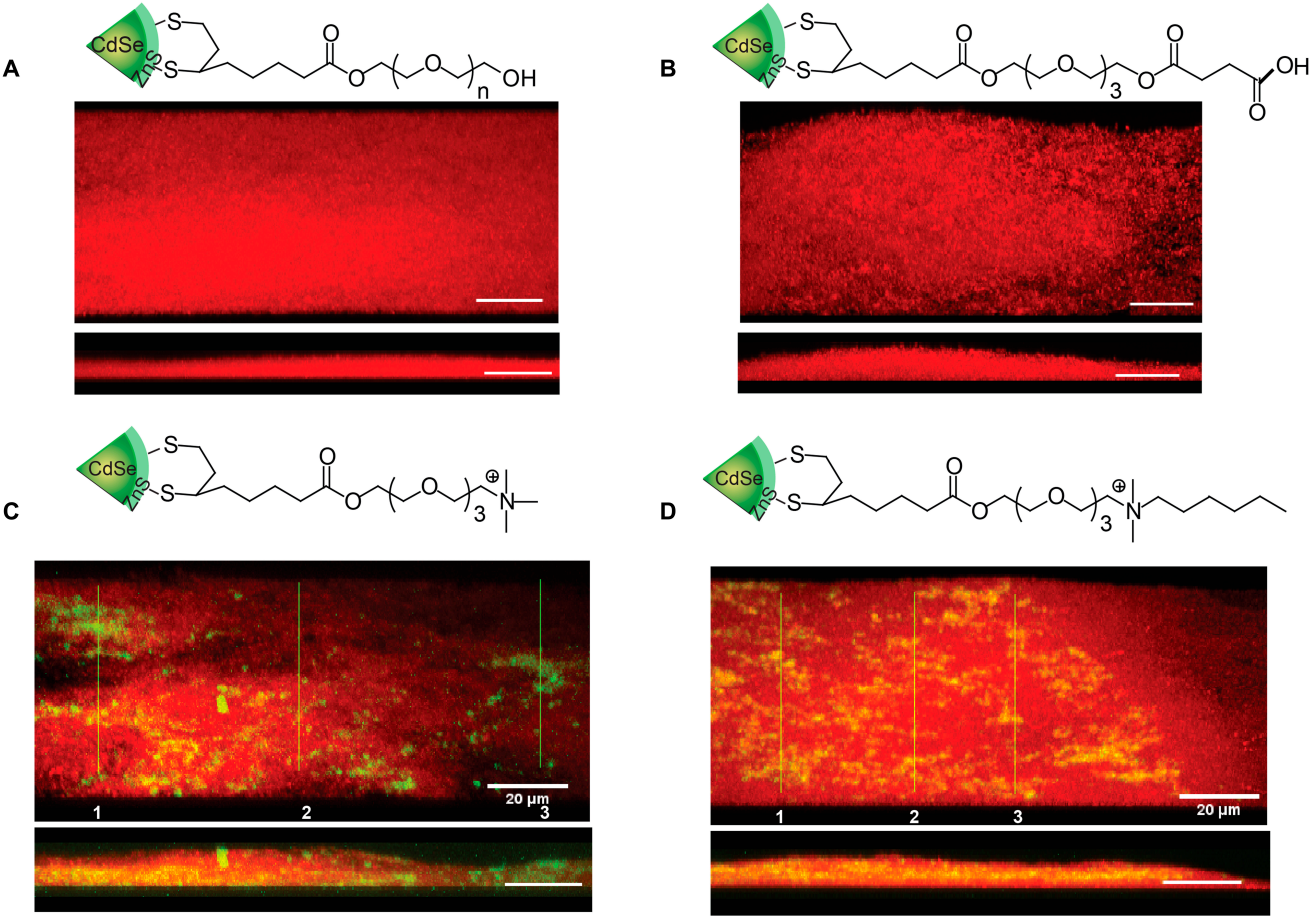
Penetration of quantum dots (green) into red fluorescent protein-expressing *E. coli* biofilms. Micrographs indicate that (A) neutral and (B) anionic QDs do not interact with the biofilm. (C) Hydrophilic cationic nanoparticles do not co-localize with cells, indicating a through-EPS transport process. (D) Hydrophobic QDs co-localize with cells, suggesting a cell-to-cell transport process. Adapted by the author from Ref. [50], Royal Society of Chemistry.

The overall negative charge of the biofilm suggests that charge will be a strong factor for the interaction of NPs with biofilms. Quantum dots (QDs) of ~10 nm diameter were used to probe the role of charge in NP–biofilm interactions [50]. Predictably, anionic QDs did not interact with the biofilms (electrostatic repulsion with the anionic bacteria and EPS), with neither attachment nor penetration observed. Neutral QDs likewise were non-interacting (no driving force for interaction). Cationic QDs can be predicted to attach to the anionic biofilm; however, transport through this “sticky” environment can be difficult to predict. It turns out that both hydrophilic and hydrophobic cationic QDs are readily transported into biofilms. Intriguingly, this process appears to occur via 2 different mechanisms. Hydrophilic QDs localize in the ECM, suggesting transport through this pathway. In contrast, hydrophobic QDs localized in cells, consistent with an “island hopping” mechanism that proceeds through the bacteria (Fig. 2).

**Fig. 3.**
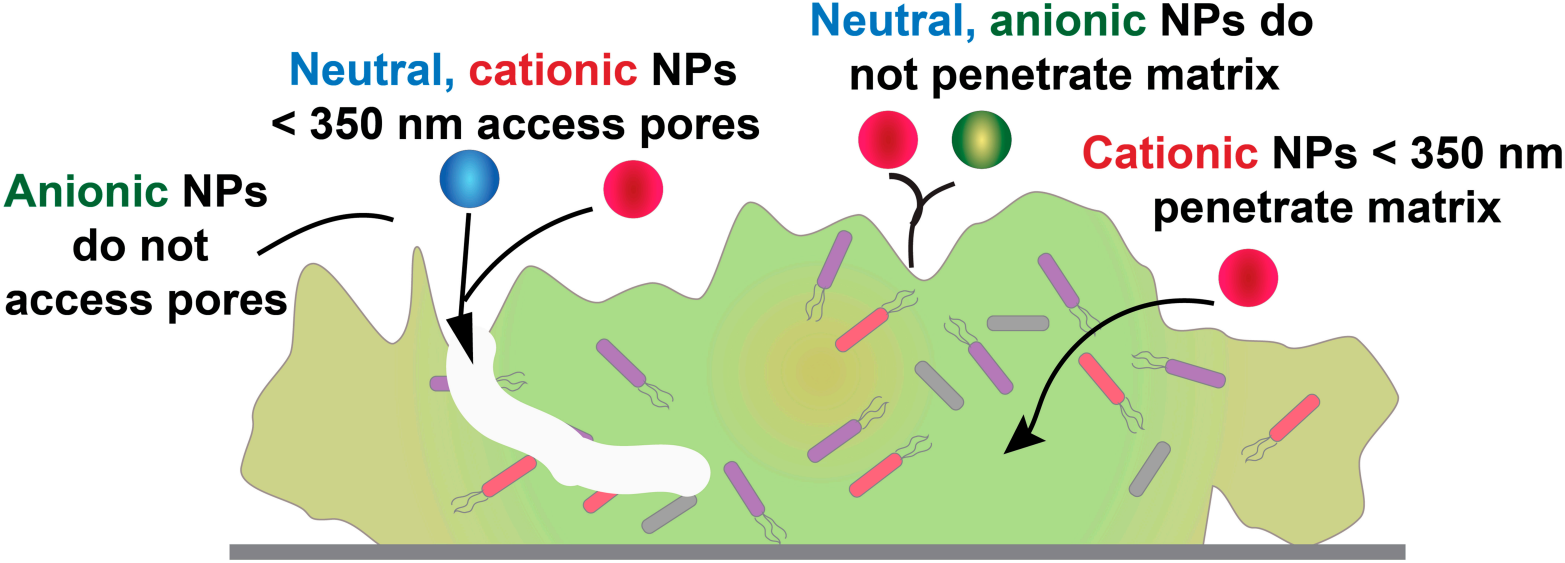
General design principles for designing nanomaterials to penetrate biofilms.

Size presents another key determinant for NP–biofilm interactions. Water pores in biofilms come in a range of diameters. Studies show that uncharged NPs <350 nm are able to access much of the biofilm depth though the water channels, providing an upper limit for uncharged particles [51,52]. Given that transport along a pore would be expected to be much faster than through the biofilm, the 350-nm scale provides a good target for cationic particles as well (Fig. 3).

## Nanomaterials as Therapeutics against Biofilms

Penetration of nanomaterials into biofilms brings to bear all the tools described above for killing of planktonic bacteria to the treatment of biofilm infections. The ability to access embedded cells (and in particular persister cells) provides a promising strategy for overcoming both the physical barriers presented by the EPS and the challenges arising from phenotypic diversity in biofilms.

### Disruption of biofilms using nanomaterials

As described above, cationic nanomaterials have the ability to penetrate into biofilms. This penetration can occur with disruption of the EPS, providing a means of completely disrupting biofilms. Then, cationic nanomaterials can kill the resident bacteria through membrane disruption [53]. These capabilities make nanomaterials a “one-stop shop” for the treatment of biofilm infections [54].

**Fig. 4.**
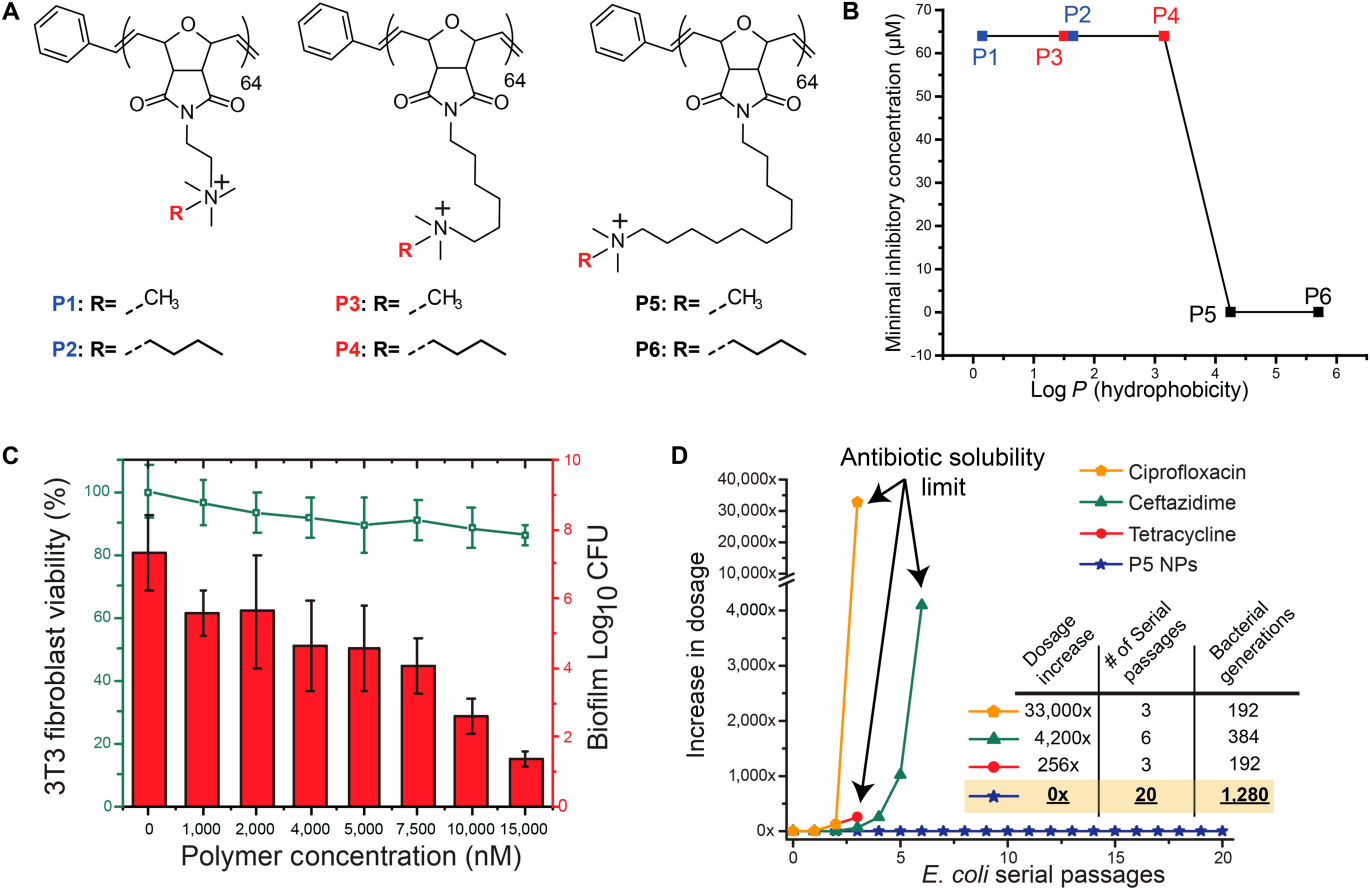
Effective and selective killing of biofilms. (A) Structures of cationic poly(oxanorbornene) polymers. (B) MIC values of polymers against *E. coli* (CD-2). Log *P* is the calculated hydrophobic values of the corresponding monomers. (C) Viability of *E. coli* biofilms and the 3T3 fibroblast cell co-culture model after incubation (3 h) with P5 NPs. (D) Resistance development during serial passaging in the presence of sub-MIC levels of antibiotics, and lack of inhibition observed with P5 nanoparticles. Adapted from Ref. [56]. ©2018 with permission of the American Chemical Society.

An example of penetration of biofilms and killing of resident bacteria killing is provided by poly(oxanorborneneimide) [55] polymers self-assembled into cationic NPs (Fig. 4A) [56]. This system provides an example of a cationic amphiphile with hydrophobicity tuned by the alkyl spacer on the quaternary ammonium side chain. With this family, amphiphilic polymers with a greater amount of hydrophobicity provided effective killing of bacteria (Fig. 4B). These NPs provided an antimicrobial with high efficacy against bacterial biofilms (including *P. aeruginosa* and methicillin-resistant *Staphylococcus aureus* [MRSA]). Of equal importance, high selectivity was observed for these NPs toward bacteria relative to mammalian cells (Fig. 4C). Perhaps most importantly, no resistance was observed with the polymer NPs during serial passaging, in stark contrast to that observed with antibiotics (Fig. 4D). This lack of resistance development suggests that this is one of the bactericidal pathways that nanomaterials can access that bacteria do not have defenses for.

**Fig. 5.**
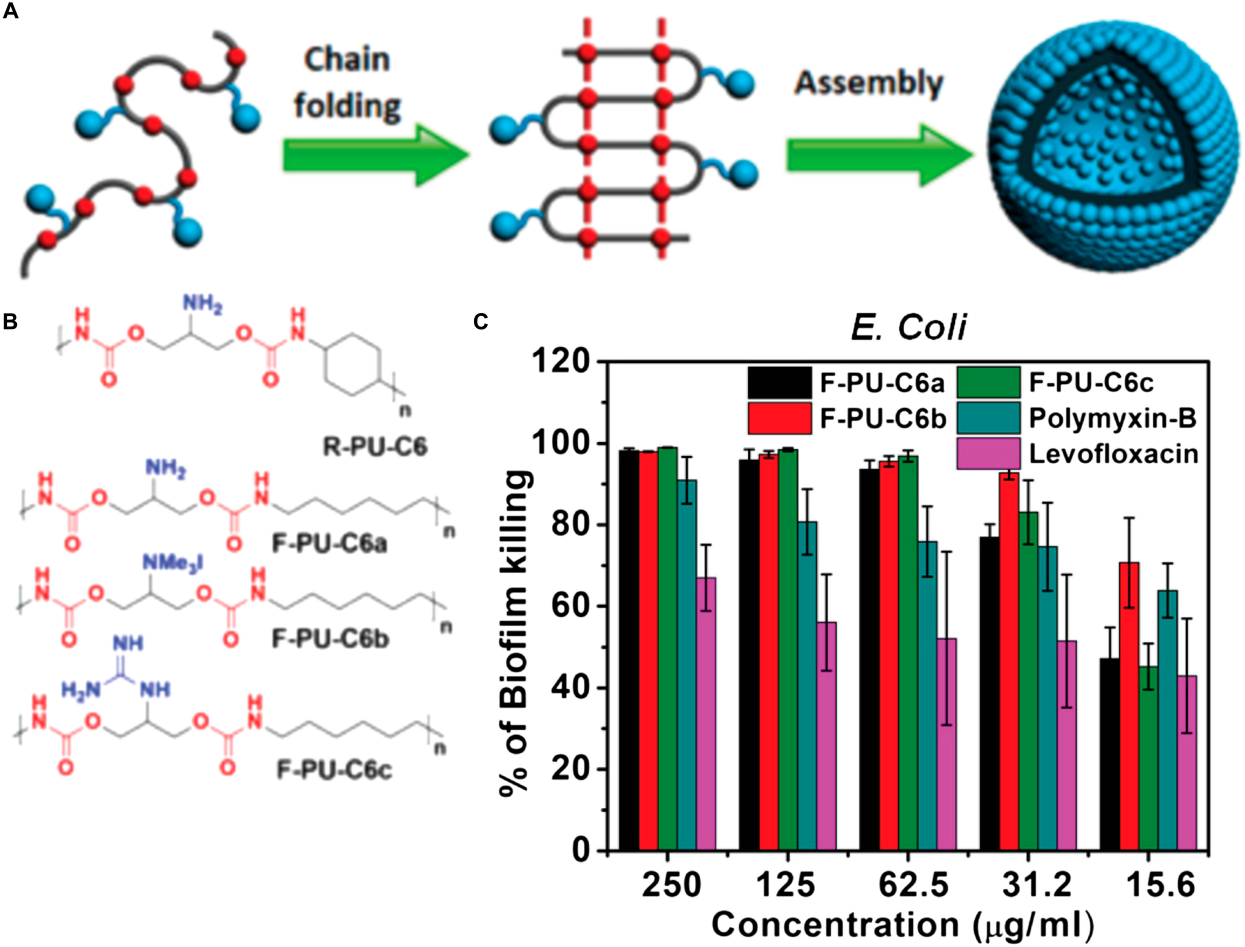
(A) Chain-folding of polyurethane polymers to provide self-assembled nanostructures. (B) Polyurethanes featuring rigid backbones (R-PU-C6) do not fold, aggregate, and are inactive against bacteria. (C) Polyurethanes with flexible backbones fold and effectively kill bacteria in biofilms. Adapted from Ref. [57] with permission of the Royal Society of Chemistry.

The structure provided by nanomaterials is an important determinant of their activity against biofilms. Barman et al. [57] developed a family of polymers featuring alternating co-polymers of polyurethane (Fig. 5A). This study showed that chain-folded structures generated from systems with flexible linkers F-PU-6a-c (Fig. 5B) provided effective killing of bacteria, whereas polymer F-PU-6 with a rigid backbone was not effective due to lack of stable nanoassembly formation. Efficient killing was demonstrated for biofilms of Gram-positive *S. aureus* and Gram-negative *Escherichia coli* (Fig. 5B).

### Delivery of antimicrobials into biofilms

**Fig. 6.**
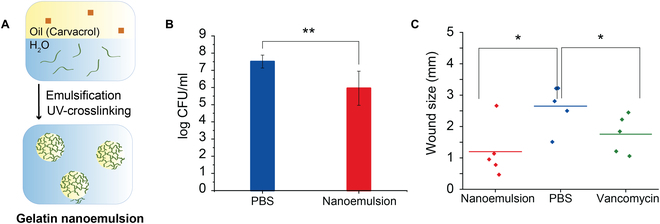
(A) Fabrication of carvacrol-loaded gelatin nanoemulsions through emulsification and riboflavin-mediated UV-crosslinking. Gelatin nanoemulsions were tested against a 4-day-old MRSA biofilm in a mouse wound model. (B) Nanoemulsion reduces bacterial load as shown by colony counts from the infected wounds. (C) Wound healing is enhanced by the gelatin nanoemulsions after 4 days with one treatment per day. Adapted by the author from Ref. [61], Royal Society of Chemistry.

Nanomaterials have the capability of delivering therapeutics deep into biofilms. This penetration imparts antibiofilm activity to therapeutics that would not normally penetrate the biofilm matrix [58]. An example is provided by polymer-based essential oil delivery. Essential oils are used by plants to resist bacterial infection, and are another front on the antimicrobial war [59]. The hydrophobicity of these oils, however, inhibits their penetration into the highly charged EPS. Nanomaterials provide an effective tool for essential oil delivery [60]. Integration of these oils into nanoemulsion “nanosponges” provides efficient delivery into biofilms. As an example, carvacrol (from oregano) was incorporated into a crosslinked gelatin nanosponge (Fig. 6A) that effectively disrupted biofilms and killed resident bacteria [61]. This system was effective against multiple species of biofilms in vitro, and reduced bacterial load (Fig. 6B) and enhanced wound healing (Fig. 6C) in a 4-day established MRSA wound biofilm in a mouse model.

Silver ions (Ag+) are highly effective at killing bacteria [42]. These ions are quite "sticky" and have difficulty penetrating into biofilms. Silver nanoclusters and NPs provide a strategy for the delivery of Ag+ [62]. Incorporation of these particles into biofilm-penetrating nanomaterial featuring a polymethacylate matrix functionalized with sugars and cationic amines enables treatment of the corresponding biofilms in vitro [63].

**Fig. 7.**
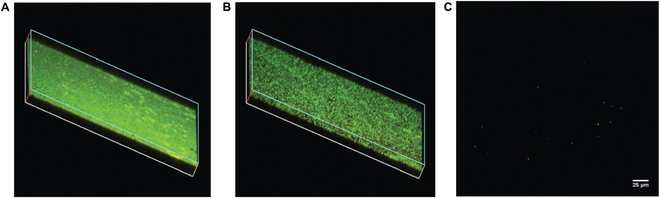
Confocal imaging of MRSA biofilms after treatment with 70-nm Fe_3_O_4_ nanoparticles (10 mg/ml) for 15 min. (A) No magnetic field (control); (B) partial disruption from heating of NPs in an AC magnetic field; and (C) complete disruption of biofilm from mechanical disruption through treatment of NPs with a rotating magnetic field. Adapted from Ref. [66] with permission of the Royal Society of Chemistry.

Localized activity is an important strategy for increasing efficacy while minimizing off-target effects. Nanomaterials have unique physical properties that can be used to enhance delivery into biofilms with spatial control. Superparamagnetic NPs (i.e. magnetic particles with easily flipped dipoles) can be heated using an alternating magnetic field [64]. This magnetic field heating was used to enhance the penetration and therapeutic efficiency of superparamagnetic iron oxide particles coated with silver rings [65]. In a related study, an alternating magnetic field was likewise used to disrupt biofilms treated with 70-nm Fe_3_O_4_ particles (Fig. 7) [66]. Far greater disruption, however, was observed using a rotating magnetic field that mechanically perturbed the biofilms. These modes of magnetically controlled biofilm disruption provide strategies that could readily be co-deployed with a wide range of other therapeutic agents.

**Fig. 8.**
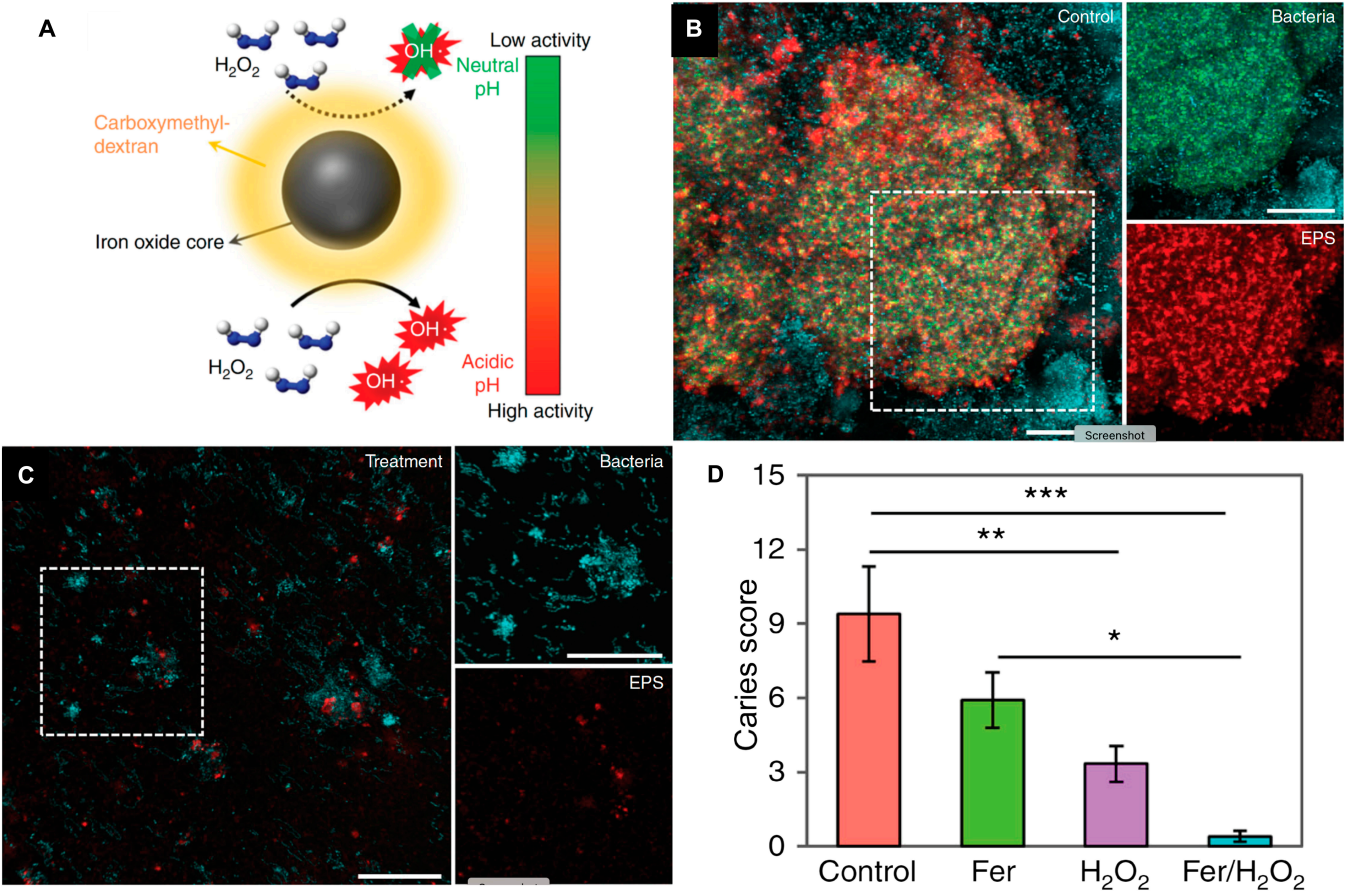
Ferumoxytol nanozymes are effective against oral biofilms. (A) Activation of ROS generation at low pH. (B) Confocal imaging of biofilms from pooled human plaque sample showing vehicle-control-treated biofilm. (C) Biofilm treated with ferumoxytol/H_2_O_2_ to generate ROS in situ*.* Total bacteria (blue), *S. mutans* cells (green), and EPS (red); scale bars: 50 μm. (D) Therapeutic efficacy of topical ferumoxytol/H_2_O_2_ against tooth decay in a mouse model (*S. mutans* UA159). Graph shows the number of moderate caries after a daily regimen of treatment for 22 days. Similar results were observed for initial and extensive lesions. Adapted from Ref. [70] under Creative Commons Attribution 4.0 International license.

Catalytic generation of antimicrobial agents at infection sites provides another strategy for localization of antimicrobial activity. Biomimetic nanocatalysts (nanozymes) provide versatile “drug factories” for in situ generation of antimicrobial species [67]. The most common embodiment of nanozymes mimics the activity of catalase enzymes, activating hydrogen peroxide to ROS species [68]. Iron oxide (Fe_3_O_4_) efficiently generates ROS from peroxide and was used to treat *Streptococcus mutans* biofilms in vitro and in vivo [69]. Strategic use of the Food and Drug Administration-approved nanotherapeutic dextran-coated Fe_3_O_4_ NP ferumoxytol for ROS generation (Fig. 8A) moves this strategy substantially closer to clinical use [70]. In this work, effective elimination of biofilms was demonstrated ex vivo (Fig. 8B and C) and prevention of caries was established using a mouse model (Fig. 8D).

**Fig. 9.**
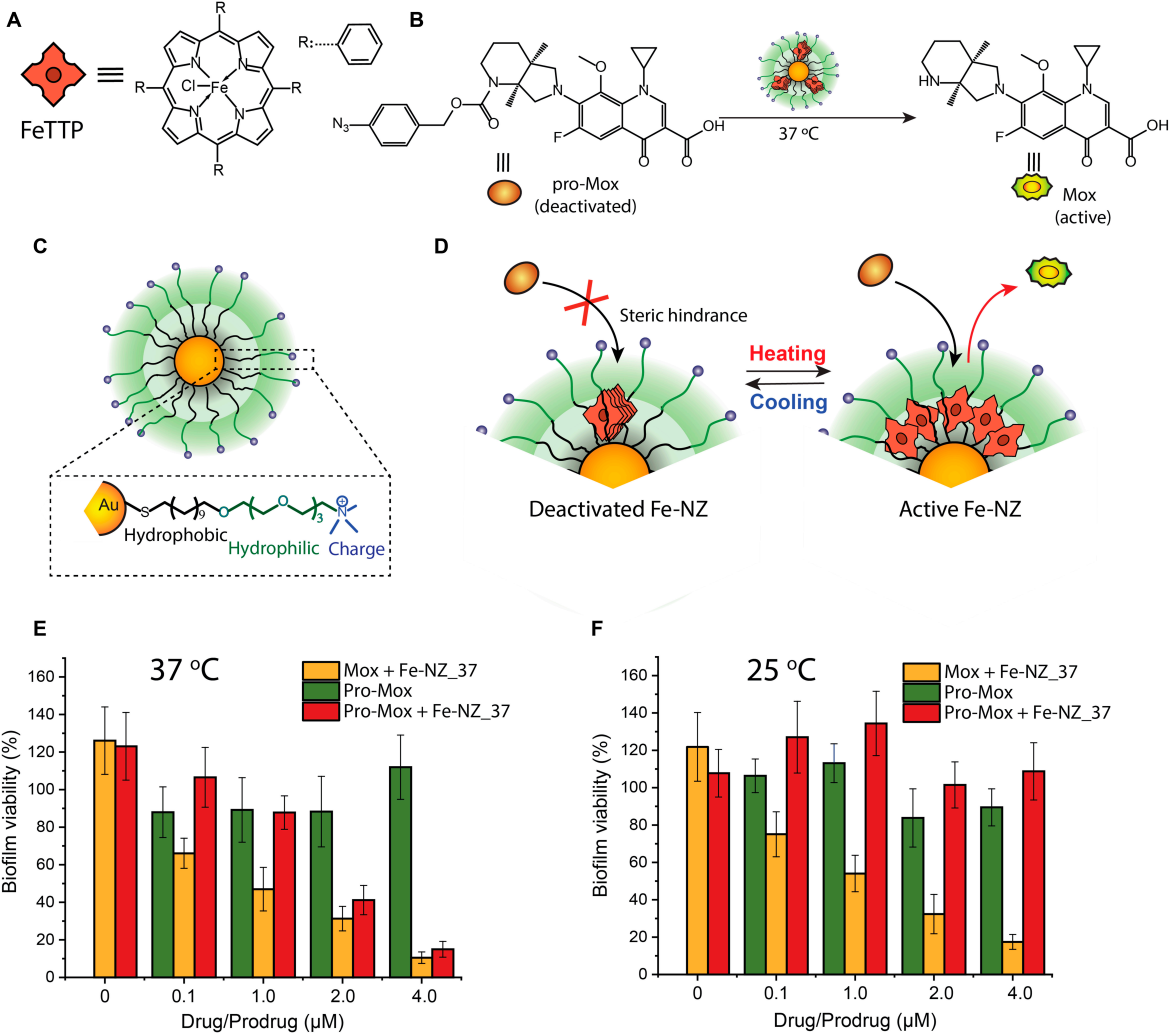
Thermally gated bioorthogonal nanozymes. (A) Structure of the FeTPP catalyst. (B) Conversion of pro-antibiotic pro-Mox to moxifloxacin (Mox) through the FeTPP-catalyzed reduction of the aryl azide. (C) Nanoparticle AuTTMA used to encapsulate FeTPP. (D) Reversible thermal gating of catalysis through controlled aggregation of FeTPP. Gating temperatures were from 25 °C (Fe-NZ_25) to 37 °C (Fe-NZ_37) with 3 °C steps. (E) *E. coli* biofilms treated with pro-Mox and Fe-NZ_37 showing the effective killing of biofilms at 37 °C. (F) At 25 °C, no pro-Mox activation occurs with Fe-NZ_37 and concomitantly no biofilm killing was observed. Adapted by the author from Ref. [76], Cell Press.

Bioorthogonal catalysis employs reactions inaccessible to natural enzymes, opening new chemical pathways for in situ generation of therapeutics [71,72]. The use of NP scaffolds for bioorthogonal catalysis can be used to stabilize reactive catalysts [73,74] and impart useful properties such as biofilm penetration [75]. Antibiofilm activity has been demonstrated using a number of different nanozyme platforms. As an example, thermally gated iron porphyrin/AuNP nanozymes were used to generate antibiotic inside biofilms, effectively killing bacteria and disrupting the biofilm [76]. Notably, antibiotic activation and bactericidal activity could be controlled by temperature. Using the appropriate nanozyme, no antibiotic was generated and no killing was observed at ambient temperature (25 °C), whereas effective prodrug uncaging and antimicrobial and antibiofilm activity occurred at 37 °C (Fig. 9).

## Summary and Outlook

MDR bacteria have evolved to survive a wide range of chemical threats, using both chemical (drug resistance) and physical means (biofilms). Nanomaterials provide us with a full toolkit of novel (to bacteria) threats that can be employed against these pathogens. Importantly, nanomaterials can access bactericidal pathways for which bacteria have not developed resistance mechanisms. The size and surface properties of NPs can be engineered to provide antimicrobial activity that can be coupled with the ability to penetrate into biofilms. This biofilm penetration capability can be used to enhance other therapeutic strategies, including both direct antimicrobial delivery and in situ therapeutic generation using nanocatalysts. Full use of these capabilities will require selectivity for pathogens relative to the host cells, a requirement that is being addressed as the field evolves. Additionally, there are a number of emerging areas where nanotechnology can be used to treat biofilm infections, including inhibition of EPS matrix formation [25] and interruption of quorum sensing [77].

The challenges in bringing antibiofilm nanomaterials to the clinic are the same as for other nanotherapeutics. The in vivo activity and safety of nanomaterials is harder to predict than for small molecules, in large part due to the rich diversity of structures possible with nanomaterials. This barrier will be diminished as our understanding of nanomaterial behavior in vivo increases. The hurdle can also be avoided through use of already approved therapeutics [70] and diminished through the use of “safe” components for nanomaterial fabrication [61]. Taken together, we can hope that nanomaterials will enable us to avoid the predicted catastrophe caused by “untreatable” MDR biofilm infections.
